# Vulnerability of long-range inputome of basal forebrain in normal aging mice

**DOI:** 10.3389/fnagi.2025.1573906

**Published:** 2025-07-23

**Authors:** Tingting Sun, Jiale Chen, Bimin Liu, Anan Li, Hui Gong, Xiangning Li

**Affiliations:** ^1^Wuhan National Laboratory for Optoelectronics, Britton Chance Center for Biomedical Photonics, Huazhong University of Science and Technology, Wuhan, China; ^2^Key Laboratory of Biomedical Engineering of Hainan Province, School of Biomedical Engineering, Hainan University, Haikou, China; ^3^HUST-Suzhou Institute for Brainsmatics, JITRI, Suzhou, China

**Keywords:** basal forebrain, long-range input, normal aging, whole brain, three-dimensional

## Abstract

**Introduction:**

As the human undergoes the process of aging, it becomes evident that the elderly population exhibits age-related cognitive decline. The basal forebrain (BF) has been shown to have complex connections with the hippocampus (Hip) and medial prefrontal cortex (mPFC) through circuits, and is involved in cognitive functions. However, which circuit is most vulnerable during normal aging remains unclear.

**Methods:**

Utilizing a combination of viral tracing and fluorescence Micro-Optical sectioning tomography (fMOST), we performed quantitative analyses on the whole-brain inputs of the BF, Hip, and mPFC during normal aging.

**Results and discussion:**

The long-range inputome revealed that the nucleus of the diagonal band (NDB) of BF was vulnerability to damage, especially the connection strength of the vCA3-NDB circuit is significantly reduced, which may be related to decision making. A comparison of the 3D continuous data of BF subregions revealed that aging resulted in a weakened connection strength between each region and the olfactory areas (OLF), which obeyed a topological relationship, which might be related to the learning and memory. These results provide an anatomical foundation for understanding the selective vulnerability of BF circuit during normal aging and offer a novel perspective for future research into the treatment of age-related cognitive decline.

## Introduction

During the process of aging, there is a marked degradation of cognitive function, which is usually accompanied by an observable reduction in the speed of information processing that is not depend on executive function. The normal aging is associated with degenerative diseases, and the age is a risk factor for some neurodegenerative diseases (Hou et al., 2019; Ng and Zetterberg, 2025). Therefore, actively exploring the circuit mechanism of normal aging is of great significance for the prevention and treatment of neurodegenerative diseases.

Cognitive function involves the cooperation of many brain regions, of which the basal forebrain (BF) plays a crucial role in both cognitive function and degenerative diseases (Tian et al., 2022; Chaves-Coira et al., 2023). The BF is a complex structure with multiple subregions, including the medial septal nucleus (MS), NDB and substantia innominata (SI), which related to attention, memory storage and retrieval (Ananth et al., 2023). Structural magnetic resonance imaging studies have showed that the atrophy of the BF is evident in both elderly individuals (Shi et al., 2024) and the patients of Alzheimer’s disease (AD; Xia et al., 2024), indicating that BF undergoes degenerative changes with the increase of age, which involves neuronal damage and the degeneration of circuit structure. Among patients with AD, the degeneration of axonal myelin sheaths between the BF and the upstream and downstream brain regions, which will slow down the signal transmission speed (Ren et al., 2023). Among the AD model mice, the axonal swelling of BF neurons had increased, which is not conducive to the release of neurotransmitters (Sun et al., 2022). Therefore, these age-related changes of BF have an adverse effect on the integration of information across brain regions, which will inevitably hinder the execution of cognitive functions.

The major interconnected brain regions of the BF, including the hippocampus (Hip) and medial prefrontal cortex (mPFC), are also associated with cognitive function. The Hip plays a role in cognitive functions beyond episodic memory, such as processing relevant information in working memory (Deng et al., 2025). The structural integrity of the Hip is essential for normal learning and memory consolidation (Shi et al., 2023), and Hip atrophy is thought to be the culprit of age-related cognitive impairment (Reuben et al., 2011; Hu and Li, 2020). Studies showed that the shrinkage of Hip volume and the delayed response time to stimuli are indicative of a slower processing of information (Papp et al., 2014). As the main subregion of the Hip, the CA3 volume shrinkage indicates the damage to the circuit structure (Zhang et al., 2020) and the functional network (Nyberg et al., 2019). The mPFC also plays an important role in regulating many cognitive functions (Ma et al., 2025). In neurodegenerative diseases, alterations in the mPFC network have been reported to be associated with cognitive decline (Allen and Morishita, 2024), possibly as a direct result of aging (Jobson et al., 2021), a speculation supported by both human (Chow et al., 2022) and primate (Cramer et al., 2018). The prelimbic area (PL), as a primary subregion of mPFC, is closely associated with age-related cognitive dysfunction. Impairment of PL not only affects the flexible transformation of behavior but also cognitive functions such as spatial memory (Schoenfeld et al., 2014).

The NDB, CA3 and PL are all implicated in age-related cognitive impairment. It is well established that the transmission of signals between brain regions, that is, the integrity of the circuit structure, is critical for the execution of functions. The presence of age- related cognitive impairment may be indicative of a structural abnormality within the circuitry that connects these three brain regions. However, the question remains unanswered as to whether simultaneous damage occurs to circuits of the three brain regions during normal aging. Studies have shown that age-related cognitive dysfunction may reflect the failure of input regulation of a certain brain region (Somera et al., 2023). Although there may be mutual projection between the NDB, CA3 and PL, the input circuit can be used to represent the connections between brain regions. Furthermore, counting the number of input neurons can help to quickly identify the damaged brain region and circuit.

The prevailing focus of contemporary research on circuit aging is centered on the microscopic level of intracellular molecules and the macroscopic level of functional imaging of the brain *in vivo*. However, there is a paucity of research addressing the mesoscopic level of 3D continuous datasets of aging with circuit structure. Array fluorescence Micro-Optical sectioning tomography (Array-fMOST) has established a comprehensive system, enabling rapid imaging of multiple samples (Chen et al., 2023). To obtain the 3D coordinates of the input-neurons, the MOST team further developed the Brain-wide Positioning System to realize the accurate positioning of the spatial position of a single cell (Gong et al., 2016).

In this study, viral tracer combined with Array-fMOST was employed to obtain 3D continuous input datasets of aging. By comparing the changes of input pattern of PL, NDB and CA3 during normal aging, we found that NDB subregion of BF has its particularity, the long-range inputome of NDB showed vulnerability to damage during aging, especially in vCA3-NDB circuit. This study aims to delve deeper into the alterations of input patterns within different subregions of the BF during the process of aging. The 3D continuous data revealed a reduction on input neurons of the BF different subregions, which corresponded topologically to olfactory areas (OLF). These results provide provides anatomical data for the treatment of age-dependent cognitive impairment.

## Materials and methods

### Animals

According to the representative age ranges of C57BL/6J mice described by Flurkey et al. (2007), mice aged 3–6 months (Young group, *n* = 15) and 18–24 months (Old group, *n* = 15) were selected. All C57BL/6J mice (Jackson Laboratory, Bar Harbor, ME, USA) were initially pair-housed on a 12-h light/dark cycle with standard laboratory chow and water freely available. The physiological conditions of these mice were normal, and no severe hair loss or tumor growth occurred. And the animal experiments were approved by the Animal Ethics Committee of the Huazhong University of Science and Technology.

### Virus injection

For retrograde tracing, the virus we used is the RV-N2C(G)-ΔG-EGFP (Wuhan BrainVTA Co., Ltd., China). According to a large number of published research (Zhu et al., 2020; Chen et al., 2023), we waited a week for the virus to express. The 50 nl of RV-N2C(G)-ΔG-EGFP (3.00E + 08 IFU/mL) was injected into the PL (bregma, 2.22 mm; lateral, 0.3 mm; ventral, −2.0 mm), MS (bregma, 0.98 mm; lateral, 0.0 mm; ventral, −4.25 mm), NDB (bregma, 0.62 mm; lateral, 0.7 mm; ventral, −5.45 mm), SI (bregma, −0.58 mm; lateral, 1.75 mm; ventral, −4.8 mm), CA3 (bregma, −2.15 mm; lateral, 2.35 mm; ventral, −2.25 mm) of adult and old mice, respectively. After the surgery, the incisions were stitched and lincomycin hydrochloride and lidocaine hydrochloride gel was applied to prevent inflammation and alleviate pain for the animals.

### Agarose embedding

The prepared mouse brain was embedded in agarose. The brains were dried and embedded in melted oxidize agarose using a silicone mold. For multiple samples, we set samples in an array arrangement (Chen et al., 2023). The brains were placed in a 55°C water bath until the surfaces of the brains were fully coated with agarose. During the water bath, the orientation of the brain could be easily adjusted to ensure the appropriate sectioning angle for the whole brain. Then, the brains were left at room temperature for 0.5 h to allow the agarose to solidify.

### Array-fMOST for whole-brain imaging

The imaging system is composed of two primary components: microscope and microtome (Chen et al., 2023). The optical microscope is composed of line scanning confocal microscope. Fluorescence signals excited by two lasers at 488 nm and 561 nm are received by two sCMOS cameras through an array of optical elements for rapid acquisition of green and red dual-channel data. The slicing device is composed of self-made high-precision vibrating slicing machine. In the process of data acquisition, when multiple scanning image strips can cover the entire sample surface, the vibrating microtome slices according to the section 3 μm thickness, which is set by the program. This image-slice-imaging cycle is repeated until all the data for the sample is obtained, with a three-dimensional voxel resolution of 0.65 μm × 0.65 μm × 3 μm.

### Data processing and registration

The stripe-shaped raw data were initially assembled together into entire coronal section images. Then the shading was removed by illumination correction (Ding et al., 2013). The gray value of pixels in each row were then aggregated to form a signal series. The series was smoothed twice independently with different spans, and the ratios of smoothed series were used as illumination correction coefficient. And the collected data set, we first preprocessed the image to correct the uneven illumination and eliminate background noise (Gong et al., 2016). For cell recognition, the images were sequentially filtered with top-hat operation, binarization, and opening operations (Chen et al., 2021). The resolution of the images used in this study was 0.65 μm/pixel, therefore, we chose a disk-shaped structure element with a radius of 9 pixels for the top-hat filter and 5 pixels for the opening filter. For binarization, the threshold depends on the signal-to-background ratio and the absolute intensity of the image, and we selected 30 as the fluorescence intensity threshold. When all slices were recognized and registered, the number of labeled neurons in each region was counted automatically. Because the reorganized RV can clearly mark the neurons, we manually checked the results to eliminate imperceptible errors. Then, we warped the soma coordinates to Allen CCFv3 using the transformation parameters from the registration (Ni et al., 2020). Subsequently, we calculated the number and proportion of input neurons in each brain region of interest to generate the quantified whole-brain inputs. Finally, we extracted the signals to obtain SWC files, and visualized the data set using Amira software (v.5.2.2).

### Quantitative analysis

All statistical graphs were generated using the Prism v8.0 software (GraphPad, La Jolla, CA, USA), and the data were analyzed with multiple *t*-tests. Multiple *t*-tests was performed on data to assess the main effects of brain region and age. If significant differences were found, the False Discovery Rate was used to determine the source of the difference. The confidence level (*p*-value) was set to 0.05, and the results were presented as mean ± SEM.

## Results

### The whole-brain inputome of NDB, CA3 and PL

**FIGURE 1:**
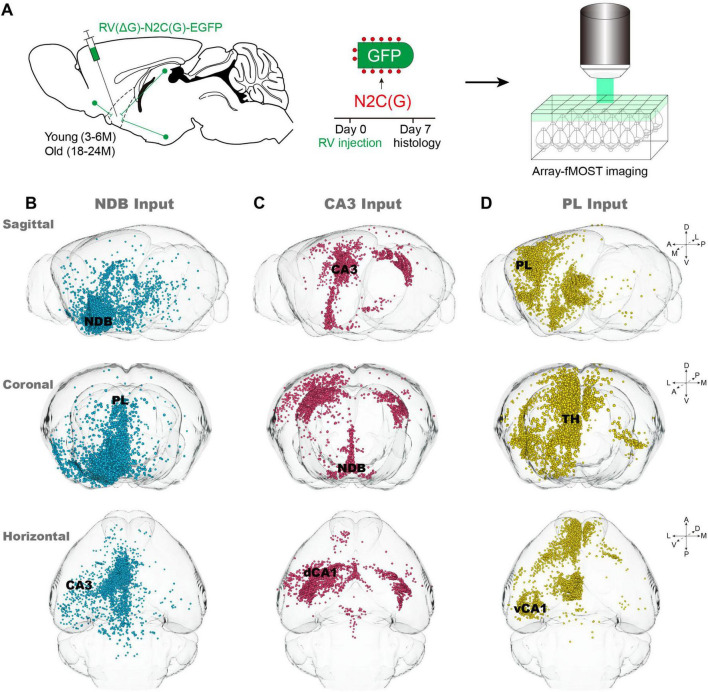
Acquisition of whole-brain input data of nucleus of the diagonal band (NDB), CA3 and PL. **(A)** Pipeline for obtaining input whole-brain data. **(B–D)** Typical sagittal, horizontal and coronal input patterns of NDB, CA3 and prelimbic area (PL) in young mice. The primarily input brain regions are indicated by black font.

According to the representative age ranges of C57BL/6J mice described by Flurkey et al. (2007), mice aged 3–6 months (Young group) and 18–24 months (Old group) were selected. Due to suboptimal surgical tolerance in aged mice, the rapidly expressed virus RV-N2C(G)-ΔG-EGFP was selected for labeling input-neurons. After 7 days of virus expression, samples were embedded in agarose, and whole-brain input data sets were obtained using Array-fMOST (Figure 1A). The utilization of RV-N2C(G)-ΔG-EGFP, which retrograde labels the soma of the neuron from the axon terminals, without marking start cells at the injection site. However, the injection site can be determined by locally input neurons from typical sagittal input patterns of NDB, CA3 and PL (Figures 1B–D). NDB exhibits a wide range of inputs distributed across the whole-brain, with input neurons located in both CA3 and PL (Figure 1B). In contrast, the distribution of input neurons of CA3 is relatively limited, being found exclusively in the ipsilateral and contralateral Hip and BF (Figure 1C). Compared with CA3, the input neurons of PL exhibit a more widespread distribution, extending not only in the local cortex and BF, but also in the thalamus (Figure 1D). A notable observation is the presence of variation in the input patterns between the NDB, CA3 and PL, such as the distribution of input neurons in the Hip. The input neurons of NDB are diffusely distributed in the Hip, which is subdivided into the dorsal and ventral regions within the Hip. However, the input neurons of CA3 are distributed in the dorsal Hip, including the dCA1, while the input neurons of PL are concentrated in the ventral CA1 (vCA1).

### The inputome of the NDB, CA3 and PL during normal aging

**FIGURE 2:**
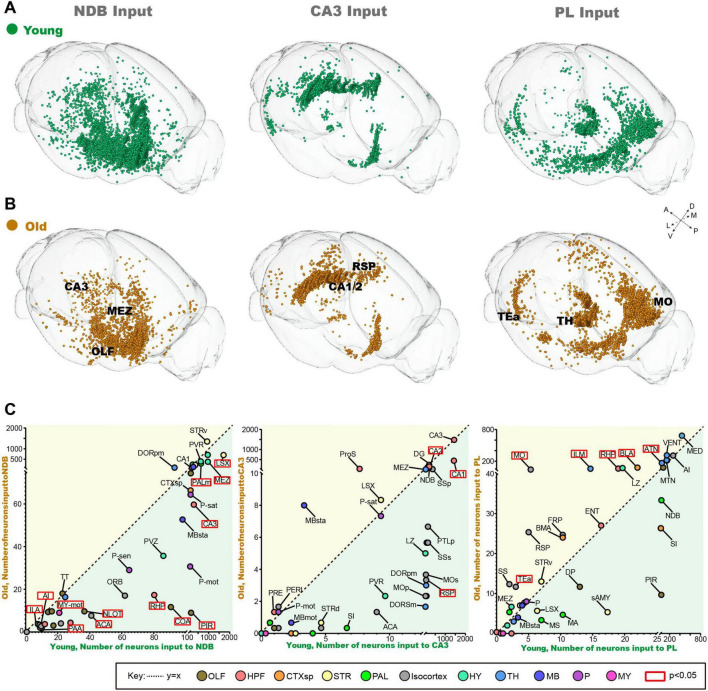
The changes of inputome of NDB, CA3 and PL during normal aging. **(A)** The 3D displays of input neuron of NDB, CA3 and PL in young mice. **(B)** The 3D displays of input neuron of NDB, CA3 and PL in old mice. **(C)** The variation trend of whole-brain input pattern of NDB, CA3 and PL during normal aging. The red boxes indicate areas with significant changes in the number of input neurons. *n* = 3. To mark the names of different brain regions, the bars have been added without meaning.

In order identify vulnerable input circuits during normal aging, we compared the changes of the whole-brain input pattern of NDB, CA3 and PL. It is noteworthy that in order to delineate the trends in the number of input neurons, the areas beneath the dotted line (y = x) are where old mice exhibit a reduction in input neurons compared to young mice (Figure 2C). The long-range inputome to NDB showed vulnerability to damage during normal aging, with the brain regions exhibiting significantly reduced input-neuron, predominantly in Hip (Figures 2A, B). Furthermore, the input areas of NDB are almost below the dotted line, and the areas with significantly reduced input are concentrated in the limbic system, such as the OLF (Figure 2C). The whole-brain input pattern of CA3 shows no significant alterations (Figures 2A–C). In the whole-brain input pattern of PL, the input neurons increased significantly in the thalamus (TH) during normal aging (Figures 2A, B). The number of input neurons increased in the dorsal thalamic nucleus (ILM) and the anterior thalamic group (ATN). In summary, our findings indicate that NDB is distinctive feature of aging, the long-range inputome to NDB showed vulnerability to damage, with areas exhibiting significantly reduced input neurons being concentrated in the Hip and OLF.

### The changes of inputome of NDB, CA3 and PL in each subregion of Hip

**FIGURE 3:**
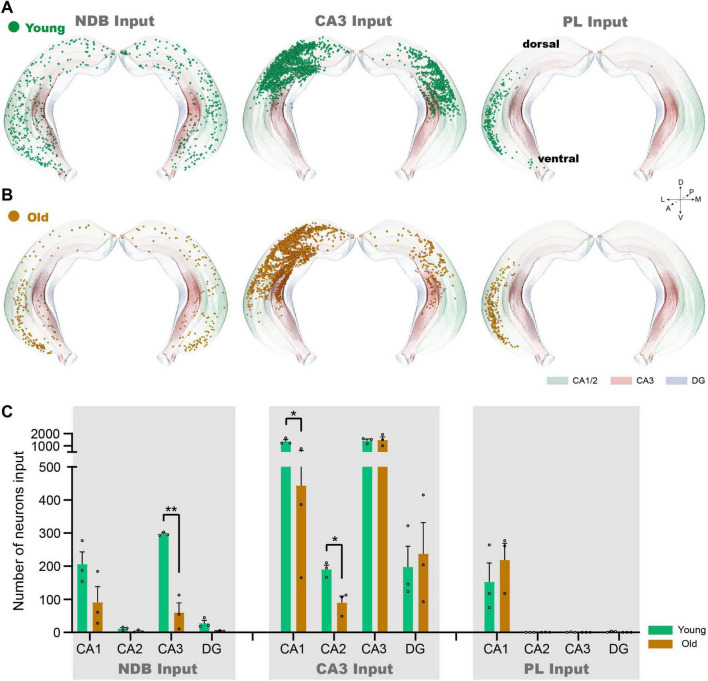
The changes of inputome of NDB, CA3 and PL in each subregion of Hip. **(A)** The 3D displays of the Hip of NDB, CA3 and PL input neurons in young mice. **(B)** The 3D displays of the Hip input neuron of NDB, CA3 and PL in old mice. **(C)** Quantitative statistics of input neurons in each subregion of the Hip during normal aging. *n* = 3. *p < 0.05, **p < 0.01.

The input neurons of NDB, CA3 and PL are distributed throughout the Hip. To further analyze the changes of input neurons in the Hip during normal aging, we further investigate each subregion of the Hip. In the conventional coronal plane of the Hip, the input neurons of NDB are dispersed in the ipsilateral and contralateral Hip (Figure 3A). During normal aging, the input pattern of NDB decreased significantly in the ventral Hip, especially in vCA1 and ventral CA3 regions (vCA3) (Figure 3B). Quantitative analysis further revealed a substantial reduction in the input neurons of NDB in CA3 (*p* = 0.0013) (Figure 3C). On the contrary, the input neurons of CA3 are distributed in the dorsal Hip, which are local input neurons (Figure 3A). The input neurons of CA3 decreased significantly in dorsal Hip, such as CA1 (*p* = 0.0161) and CA2 (*p* = 0.0141) (Figures 3B, C). Additionally, the input neurons of PL are concentrated in vCA1, and distributed exclusively on the ipsilateral side (Figure 3A). In old mice, the input neurons of PL did not change in vCA1 (Figure 3B). In summary, the aging of the circuit is not confined in the local circuit, but also affect the remote circuits. The 3D continuous input data set revealed a severe impairment in the connection strength of the vCA3-NDB circuit, indicating that the vCA3-NDB circuit is susceptible to deterioration during normal aging.

### The changes of inputome of BF subregions in OLF

**FIGURE 4:**
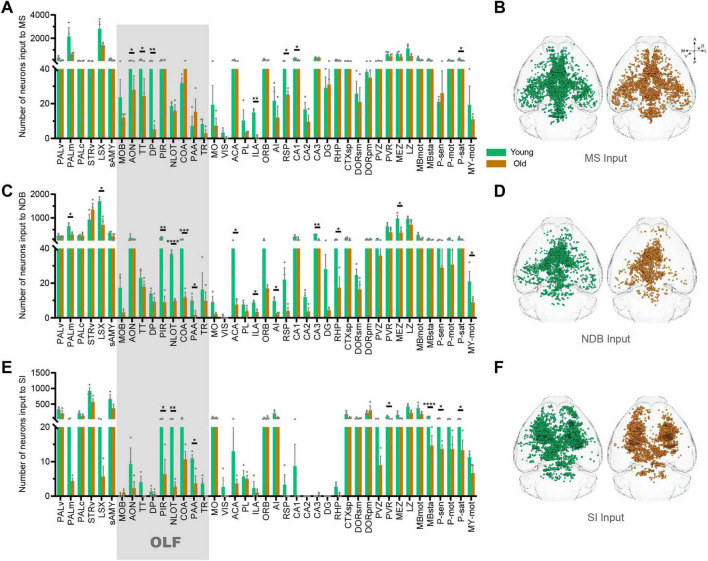
The changes of inputome of medial septal nucleus (MS), NDB, substantia innominata (SI) in olfactory areas (OLF). **(A)** Statistical analysis of input neurons of MS. *n* = 3. **(B)** The whole-brain distribution pattern of input neurons of MS. **(C)** Statistical analysis of input neurons of NDB. *n* = 3. **(D)** The whole-brain distribution pattern of input neurons of NDB. **(E)** Statistical analysis of input neurons of SI. *n* = 3. **(F)** The whole-brain distribution pattern of input neurons of SI. The shadows are subregions of the OLF. *p < 0.05, **p < 0.01, ***p < 0.001, ****p < 0.0001.

The BF contains many subregions not only the NDB, but also the anterior MS and the posterior SI. In order to further explore input changes of BF different subregions, we also obtained whole-brain input datasets for MS and SI. We found that the total number of input neurons of each subregion of BF decreased significantly in the OLF, the *p*-value of MS (*p* = 0.0397), NDB (*p* = 0.0064) and SI (*p* = 0.0423). To explore the variation patterns of input neurons within OLF, we sorted out the changes in different subregions of OLF (Figures 4A–F).

**FIGURE 5:**
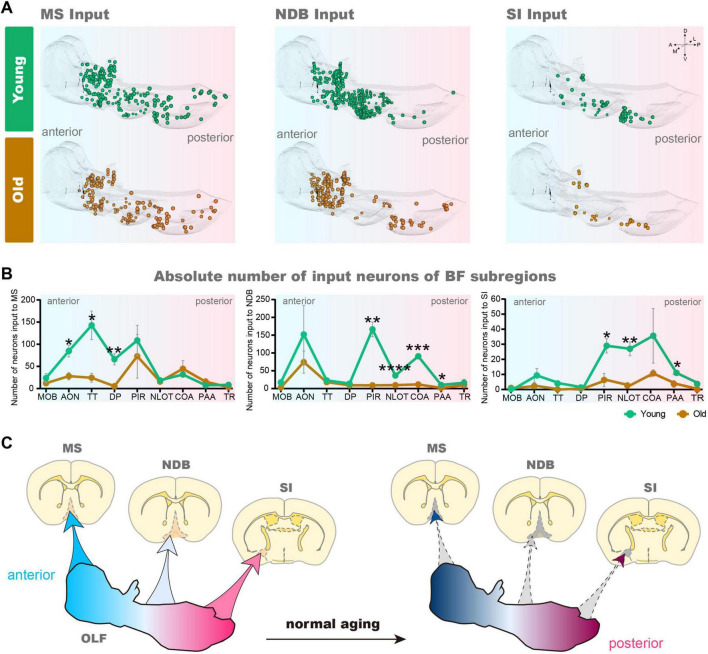
The variation of 3D distribution of input pattern of MS, NDB, SI in OLF. **(A)** The 3D displays of the OLF of MS, NDB, SI input neurons during normal aging. **(B)** Statistical analysis of input neurons of MS, NDB and SI in the OLF. *n* = 3. **(C)** Schematic diagram of changes of MS, NDB, SI input pattern in OLF. *p < 0.05, **p < 0.01, ***p < 0.001, ****p < 0.0001.

Based on the 3D distribution diagram, we found that the input neurons of MS in OLF were preferentially distributed in the anterior area, including anterior olfactory nucleus (AON), taenia tecta (TT) and dorsal peduncular area (DP). Concurrently, the input neurons in these areas were significantly reduced (AON: *p* = 0.0216, TT: *p* = 0.0247, DP: *p* = 0.0089). On the contrary, the input of NDB was predominantly distributed in the posterior OLF, including piriform area (PIR), nucleus of the lateral olfactory tract (NLOT), cortical amygdalar area (COA) and piriform-amygdalar area (PAA) (PIR: *p* = 0.0017, NLOT: *p* = 0.00009, COA: *p* = 0.0003, PAA: *p* = 0.0257), accompanied by a corresponding decrease in input neurons within these regions. Although the input of SI is not widely distributed in OLF, the areas with reduced input neurons are also concentrated in the posterior OLF (PIR: *p* = 0.0244, NLOT: *p* = 0.0072, and PAA: *p* = 0.0380) (Figures 5A, B). To sum up, there is spatial preference on input neuron distribution of BF, and the topological relationship of BF-input neurons in OLF is anterior-to-anterior and posterior-to-posterior. In the normal aging, the input changes of BF different subregions in OLF also accord with the topological relationship (Figure 5C).

## Discussion

In this study, viral tracer combined with Array-fMOST was used to obtain whole-brain input datasets of normal aging. A comparative analysis of the 3D continuous input datasets of the NDB, CA3 and PL revealed that the NDB subregion of BF exhibited particular characteristics during aging. The long-range inputome to NDB showed vulnerability to damage during normal aging, especially evident in the vCA3-NDB circuit, which was severely damaged. Furthermore, we explored the alterations of input pattern of different BF subregions normal aging. The 3D distribution diagram of the OLF exhibited a spatial preference on the input neuron distribution of different BF subregions, and the topological relationship of input neurons between BF and OLF manifested as anterior-to-anterior and posterior-to-posterior. In the aging, the changes of input pattern of BF different subregions in OLF also accord with the topological relationship.

Structural magnetic resonance results showed that BF atrophy occurred in the elderly (Shi et al., 2024), indicating that BF underwent degenerative changes, which involve the atrophy, loss of neurons and the degeneration of circuit structure. Clinical studies have confirmed that the structural damage of BF is closely related to the cognitive impairment of early progress of AD (Xia et al., 2024).

The BF is a complex structure with multiple subregions, including MS, NDB and SI. Studies have confirmed that the functions of different BF subregions are also heterogeneous. MS has been shown to regulate the extraction of spatial memory in working memory (Song et al., 2020), participate in the formation of episodic memory (Sans-Dublanc et al., 2020), and is also a key nucleus of the subcortical theta oscillation (Salib et al., 2019). NDB promotes rapid transition between non-REM and REM sleep, also plays an active role in rapidly regulating behavior, avoiding harmful stimuli (Tashakori-Sabzevar and Ward, 2018), and participating in the formation of higher-order functions such as attention and default mode network behavior (Lozano-Montes et al., 2020). SI is involved in coding aggressive behavior, dealing with aversive emotions (Zhu et al., 2021). Therefore, it is very necessary to divide into subregions and refine the circuit aging characteristics.

The aging distinctive characteristics of BF were investigated furtherly in this study. Our findings indicate that the long-range inputome to NDB of BF showed vulnerability to damage during aging. We found that the input neurons of NDB were significantly reduced in CA3, especially in vCA3, indicating that the connection strength of this circuit was severely affected by aging. BF contains various types of neurons. However, it remains unclear which type of neuron is more susceptible to the influence of upstream and downstream circuits during normal aging. In aged rats, the mRNA expression levels of choline acetyltransferase (ChAT, cholinergic) and glutamate decarboxylase (GAD67, GABAergic) both decreased, indicating that the ability of cholinergic and GABAergic neurons in BF to synthesize neurotransmitters declined (Han et al., 2002). The results of BF single-cell sequencing showed that the nuclear stress granule pathway and Double-strand break repair of 15 M mice exhibited a high degree of change in ChAT neurons, suggesting the neurons are underlying higher stress conditions, and experiencing higher levels of DNA damage (Chen et al., 2025). These results seem to suggest that ChAT neurons are more susceptible to damage during normal aging. Interestingly, more than half of the Chat neurons in BF may simultaneously express vesicular glutamate transporter type 3 (Yang et al., 2017), meaning that these neurons have a dual neurotransmitter system. Therefore, it is necessary to further subdivide the neurons in BF into subtypes to explore the vulnerability of neurons. Moreover, the existing studies have not divided CA3 into dorsal-ventral distribution for separate functional regulation. Although the circuit between the dCA3 and BF is related to spatial working memory (Song et al., 2020), while the function of the circuitry between vCA3 and BF is unclear, which may be related to the decision making (Çavdaroğlu et al., 2021).

Based on the characteristics of 3D distribution diagram, we found that there was spatial preference on input neuron distribution, and the topological connection between BF and OLF is anterior-to-anterior and posterior-to-posterior. It needs to be emphasized that the different subgroups of the same brain region have heterogeneity in cognitive functions. Studies show that the connection between NDB and anterior OLF can regulate the neural excitability, which can improve the ability of discrimination, detection and learning (Ma and Luo, 2012; Devore et al., 2014; Bendahmane et al., 2016; Zheng et al., 2018). However, blocking the muscarinic acetylcholine receptor of posterior OLF can induce the generalization of odors and impair the ability of odor recognition and learning (Wilson, 2001). These results indicate that BF play a role in the processing of olfactory information for learning and memory. Among the AD model mice, the neurons of the BF exhibit enhanced neurofibrillary tangle formation in the OLF (Lewis et al., 2001), which can be speculated that the damage to this circuit may lead to cognitive dysfunction. Our results further demonstrate that even the subregions that are adjacent in terms of spatial locations can exhibit significant disparities in their circuit structures.

A limitation of this study is the lack of analysis of different cell-type for the connection and the functional modulation. BF and the connection areas contain many types of neurons, including GABAergic, cholinergic, and glutaminergic neurons. In the future, we will conduct in-depth research on the damage of this circuit based on cell -type (Ren et al., 2025). We aim to refine the functional regulatory positions according to circuit projection preferences to provide structural data support for improving cognitive impairment. After identifying the vulnerable neuron type, we will combine multiple behavioral paradigms to provide structural evidence for rescuing age-related cognitive impairments. In addition, the retrograde tracing combined with Patch-seq (Lipovsek et al., 2021) can further explore the key genes that cause neuronal aging, providing a research direction for gene therapy.

## Conclusion

The input circuits of NDB were vulnerable during normal aging, especially the vCA3-NDB circuit. Aging resulted in weakened connection strength between OLF and BF subregions, the reduction of input pattern obeyed topological relationship.

## Data Availability

The original contributions presented in this study are included in this article/supplementary material, further inquiries can be directed to the corresponding author.
